# Conceptualizing physical activity parenting practices using expert informed concept mapping analysis

**DOI:** 10.1186/s12889-017-4487-1

**Published:** 2017-06-14

**Authors:** Louise C. Mâsse, Teresia M. O’Connor, Andrew W. Tu, Sheryl O. Hughes, Mark R. Beauchamp, Tom Baranowski, Physical Activity Parenting Expert Group

**Affiliations:** 1 BC Children’s Hospital Research Institute, School of Population and Public Health, University of British Columbia, F508-4480 Oak Street, Vancouver, BC V6H 3V4 Canada; 2 USDA/ARS Children’s Nutrition Research Center, Baylor College of Medicine, CNRC-2034 1100 Bates St, Houston, TX 77030 USA; 3 University of British Columbia, Rm 122 War Memorial Gymnasium, 6081 University Boulevard Vancouver, Vancouver, BC Canada

**Keywords:** Physical activity, Parenting practices, Children, Concept mapping, Measurement

## Abstract

**Background:**

Parents are widely recognized as playing a central role in the development of child behaviors such as physical activity. As there is little agreement as to the dimensions of physical activity-related parenting practices that should be measured or how they should be operationalized, this study engaged experts to develop an integrated conceptual framework for assessing parenting practices that influence multiple aspects of 5 to 12 year old children’s participation in physical activity. The ultimate goal of this study is to inform the development of an item bank (repository of calibrated items) aimed at measuring physical activity parenting practices.

**Methods:**

Twenty four experts from 6 countries (Australia, Canada, England, Scotland, the Netherlands, & United States (US)) sorted 77 physical activity parenting practice concepts identified from our previously published synthesis of the literature (74 measures) and survey of Canadian and US parents. Concept Mapping software was used to conduct the multi-dimensional scaling (MDS) analysis and a cluster analysis of the MDS solution of the Expert’s sorting which was qualitatively reviewed and commented on by the Experts.

**Results:**

The conceptual framework includes 12 constructs which are presented using three main domains of parenting practices (neglect/control, autonomy support, and structure). The neglect/control domain includes two constructs: permissive and pressuring parenting practices. The autonomy supportive domain includes four constructs: encouragement, guided choice, involvement in child physical activities, and praises/rewards for their child’s physical activity. Finally, the structure domain includes six constructs: co-participation, expectations, facilitation, modeling, monitoring, and restricting physical activity for safety or academic concerns.

**Conclusion:**

The concept mapping analysis provided a useful process to engage experts in re-conceptualizing physical activity parenting practices and identified key constructs to include in measures of physical activity parenting. While the constructs identified ought to be included in measures of physical activity parenting practices, it will be important to collect data among parents to further validate the content of these constructs. In conclusion, the method provided a roadmap for developing an item bank that captures key facets of physical activity parenting and ultimately serves to standardize how we operationalize measures of physical activity parenting.

## Background

Parents are widely recognized as playing a central role in the development of child behaviors that influence risk of childhood obesity [1–4]. Interventions aimed at reducing childhood obesity, have shown that the familial environment plays a significant role in enabling healthy behaviours and in influencing children’s physical activity [5, 6]. While levels of physical activity in children are known to be influenced by a range of individual, social and environmental factors, parents play a critical role in socializing their children to be physically active through their childrearing parenting styles and practices [7].

Parenting styles and parenting practices are the terms used to describe how parents communicate with their child about their behaviors. Parenting styles highlight the overarching affective childrearing behaviors that parents use to interact with their child across different contexts [8]. In contrast, parenting practices refer to content and context specific childrearing approaches parents use to bring about certain childrearing outcomes such as child engagement in physical activity [9]. Parenting practices are thought to be important for influencing child behaviors such as physical activity. Recent reviews [10–13] identified parental encouragement, modeling, co-participation, and logistic support to be associated with children’s physical activity. However, the findings across these reviews are inconsistent. Currently, little agreement exists in terms of the dimensions of physical activity-related parenting practices that should be measured or how they should be operationalized. This lack of consistency has made it difficult to compare results across studies [14] thereby limiting our ability to fully understand how parents influence children’s physical activity behaviors and how interventions may most efficiently and effectively be developed to positively influence parenting practices.

To improve comparisons across studies, one solution is to utilize Item Response Modeling (IRM) item banking which creates a repository of calibrated items. Item banking has been used to address some of the measurement challenges faced in other fields (i.e., patient-reported outcomes) including being able to compare results across studies when researchers use different measures and reducing participant burden among others [14–16]. IRM item banking supplemented with Computerized Adaptive Testing allows researchers the flexibility to select which items to include in a study while maintaining the ability to compare scores for a specific dimension across studies [15–17]. A physical activity parenting item bank requires that a conceptual framework guide the operationalization of underlying dimensions. As there is little agreement on how measures of physical activity parenting should be operationalized, [14] this study engaged researchers who have expertise in physical activity parenting practices to develop an integrated conceptual framework for assessing the practices that influence multiple aspects of 5 to 12 year old children’s participation in physical activity.

## Methods

### Participants/experts

Each expert recruited to develop the physical activity conceptual framework had to be a leading authority in: 1) developing family interventions aimed at treating or preventing childhood obesity and/or modifying health behaviors associated with obesity; and/or 2) studying the etiology of children’s obesity from the perspective of parenting and families. Experts were identified by: 1) reviewing the membership list of the International Society of Behavioral Nutrition and Physical Activity (ISBNPA) and the list of attendees of the 2012 pre-ISBNPA meeting, as it focused on improving measures of physical activity and food-related parenting practices and general parenting styles; 2) reviewing citations within recent reviews published on this topic; [11, 18] 3) conducting searches on PubMed, ERIC, PsycINFO, and ScienceDirect; and 4) talking to our network of researchers. Thirty scientists from the expert search were invited, of whom 20 participated (67% response rate) and were remunerated for devoting a day to this initiative. Four members of the research team (MRB, TB, TMO, SOH) also participated. In total, 24 experts from six countries (Australia, Canada, England, Scotland, the Netherlands, and US) provided input in conceptualizing the measures of physical activity parenting practices.

### Procedures

#### Identification of physical activity parenting practices

Concept mapping procedures traditionally involve experts in brainstorming to identify the specific constructs (in this case, parenting practices) to be included in the conceptual framework [19–21]. Given the extensive work previously conducted in this area, the physical activity parenting practices were identified by: 1) conducting a review of published measures of physical activity parenting practices and 2) collecting qualitative data through semi-qualitative interviews from 134 parents of 5 to 12 year old children to identify the practices they self-report to enable their children to be more physically active. These processes are fully described in a previous paper that examined whether current measures include practices that parents self-reported using [22]. Briefly, step one identified 74 measures which included a total of 608 items that measured physical activity parenting practices [22]. Step two identified 1378 parent responses that were coded as specific physical activity-related parenting practices [22]. In preparation for the Concept mapping procedures, this data was collected as part of the larger study and published elsewhere [22].

#### Condensing the list of physical activity parenting practices

To condense the list of physical activity parenting practices identified from the literature search and self-reported by the parents, we followed the binning and winnowing process developed by the National Institutes of Health PROMIS initiative [23]. The binning process consisted of assigning primary and secondary codes to identify items or parent responses that measured the same construct. A list of 14 primary codes (autonomy support, co-participation, encouragement, expressing negative/positive emotions, lack of parental control, logistic support/facilitation, modeling, monitoring, pressure to be active, restriction, rewards and discipline, structure of the environment, teaching & reasoning) and between 1 and 5 secondary codes (for example logistic support and facilitation included the following 5 secondary codes enrollment as well as providing equipment, financial, transportation, and general support; autonomy support included the following two secondary codes: child choosing and negotiation) per primary code were developed for this purpose [22]. Previous work by our group reported how, the 608 items from the published literature and 1378 parent responses were initially consolidated to 126 unique key parenting practices [22]. Additional work by our group to further reduce the number of physical activity parenting practice concepts for the Experts to review reduced these to 77 key physical activity parenting practices. We used a consensus process to reduce the pool of parenting practices from the literature and from parent responses where two researchers and two members of the investigative team had to agree on all decisions made in reducing the data. These key parenting practices were not measurement items, but instead captured the content from similar items that measured a similar practice. For example, one item representing a physical activity parenting practice was “Restrict [activity type] inside the house” with an example of activity type being active play, ball games, running, riding tricycle/scooter. In the literature there could have been five items linked to this parenting practice, but for the sorting task the experts were only provided the key practice and not the actual measurement items.

#### Sorting the list of physical activity parenting practices

Using the online Concept Mapping software (CS Global Max version from Concept Systems Inc., Ithaca, New York), experts were asked to sort the 77 key parenting practices identified from the published literature and parents’ responses into groups that made sense to them (i.e., aggregating similar constructs together), and name each grouping. The only restriction was to not include a miscellaneous or “junk” group, but rather to put practices that were not perceived to fit into a group or concept into its own unique group. As part of the invitation, experts reviewed the consent form for the study and were provided access to the software only if they agreed to participate which was achieved by clicking on a box.

### Analysis

Concept mapping methods developed by Kane and Trochim [24] were used to: 1) have experts sort the physical activity parenting practices identified both from the literature and parent semi-structured qualitative interviews, 2) quantitatively analyze the sorting using Multidimensional Scaling (MDS) Analysis followed by a cluster analysis, and 3) inductively develop the conceptual framework by integrating the results from the concept mapping analysis with expert input.

Groupings developed by the experts were preliminarily reviewed to determine whether the sorting was completed and whether the experts followed the instructions. One expert grouped three single parenting practices into a miscellaneous category. This group was subsequently split into three singleton groups before proceeding with the analyses as such miscellaneous groupings cannot be included in the analyses. The expert sorting was analyzed with a non-parametric MDS analysis that extracted a two-dimensional solution. The selection of a two-dimensional solution was determined a-priori as the intent of this initial processing of the data was to create a similarity matrix that could be visually displayed and interpreted. In addition, Kruskal and Wish [25] found that when MDS is combined with a cluster analysis, a two-dimensional solution is preferred. The MDS assigned an x/y coordinate to each physical activity parenting practice which was displayed on a point map and qualitatively interpreted. Parenting practices located near each other on the point map represent those that were grouped together often by the experts and thus likely measure a similar construct. Overall fit of the MDS solution is assessed by evaluating the stress value which ranges between 0 and 1. Acceptable stress values typically range from 0.205 to 0.365 when MDS is used to develop a conceptual framework [26] (as opposed to being used in controlled psychometric evaluations where lower stress values are expected) [25].

A cluster analysis of the MDS solution was then performed. Trochim’s [24] procedures were followed to identify the number of clusters retained. This iterative process started with more clusters than anticipated and sequentially reduced the number of clusters by one to examine whether the two combined clusters were conceptually similar. The procedure was stopped when it did not make conceptual sense to further combine clusters. We arbitrarily started with a 28-cluster solution as the starting point to examine all solutions with fewer clusters all the way to a 2-cluster solution. Determination of the number of clusters retained integrated the results of the cluster analysis with a qualitative analysis of the concept map, which resulted in sharpening the shape of the clusters. This process involved examining the content of each cluster and determining whether borderline parenting practices should remain in the cluster, be re-assigned to a nearby cluster, or should be moved based on conceptual knowledge. While the analytical process is quantitatively informed, there is strong qualitative component to this type of analysis as described by Trochim [24]. Importantly, the quantitative results provide the foundation for initiating the discussion among the experts but the resulting solution is qualitatively derived. Given the exploratory nature of the cluster analyses, the results aimed to identify the number of concepts to include in our conceptual framework; however, further sharpening of the content within each construct is likely to occur at the item creation stage. We a-priori opted to present the main constructs identified by the experts under the three main parenting domains of control, autonomy support, and structure as a recent expert paper highlighted its utility in the area of physical activity [14] and it utilizes a nomenclature used in the nutrition field and by developmental psychologists [9, 27–29].

Three members of the research team (AWT, LCM, & TMO), of which two were not involved in the sorting process (AWT & LCM) independently completed this process, discussed their solutions, and iteratively reviewed their solutions until the three members agreed on an initial solution. This initial solution was presented to the larger team of investigators who suggested further modification. The modified initial solution was then presented to the expert group to receive further input and ensure the experts agreed with the final solution. Having the experts review and endorse the decisions lend further credibility to this process. At all levels of the analyses, LCM and AWT took the lead in integrating the feedback received by the research team and the larger group of experts since they did not take part in the sorting. This process was followed to reduce the possibility of having a specific theory influence the selection of the clusters, although our collective knowledge of the current literature could have biased some of this process.

## Results

### Clustering of the physical activity parenting practices

**Fig. 1:**
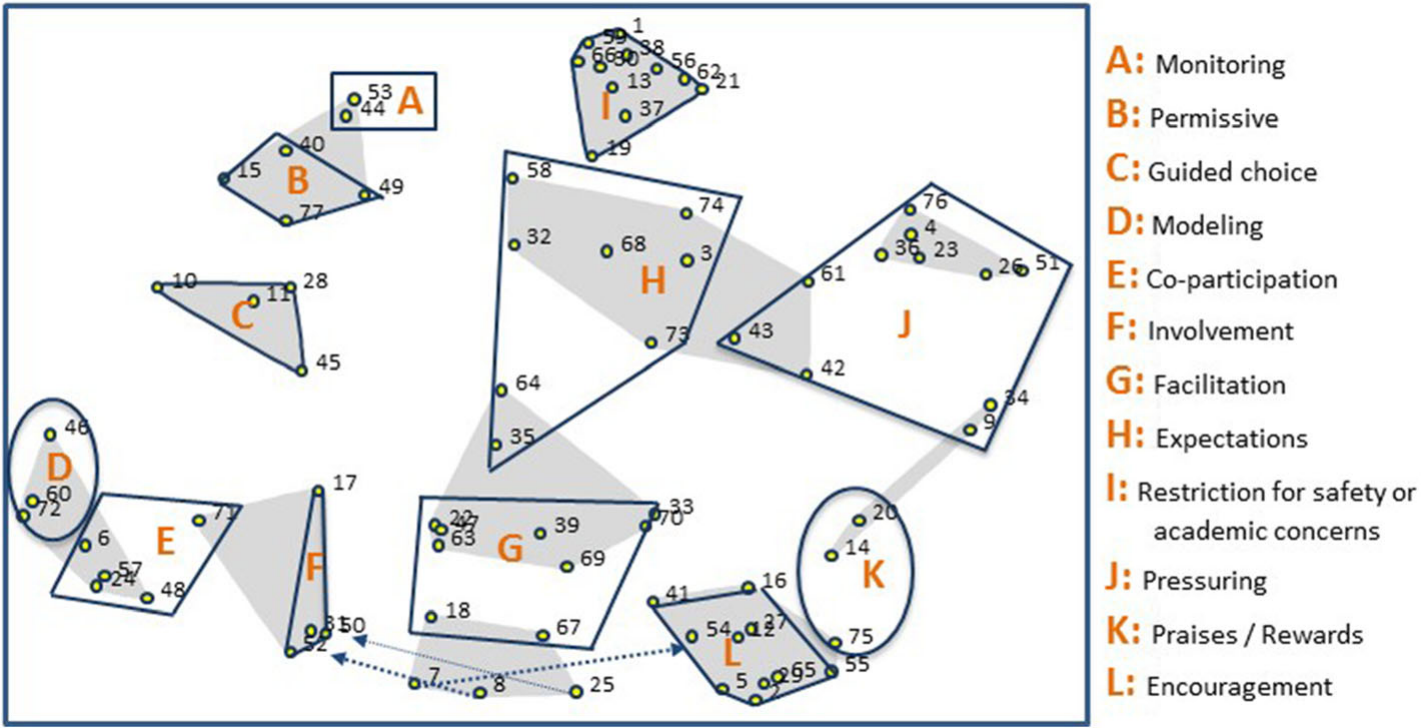
Two-dimension point map showing the 77 physical activity parenting statements clustered into 12 constructs (outlined in blue in the conceptual solution) superimposed onto the 11-cluster Multi-Dimensional Scaling solution (shaded in *grey*)

The stress value for the MDS solution was 0.285 (standard deviation of 0.04)– within the range considered acceptable for solutions used to develop conceptual frameworks [26]. The preliminary analysis conducted by three members of the research team (AWT, LCM, & TMO), initially identified seven to eight potential constructs. This preliminary solution was presented and discussed with other members of the research team (MRB, TB & SOH). Together the research team converged on a solution with 12 constructs addressing various physical activity parenting practices. After the 12 construct solution was reviewed by the expert group, further refinement and reshaping of the solution occurred. Figure 1 shows both the conceptual solution agreed upon (12 constructs are line shaped) and the 11-cluster MDS solution (shaded shapes), to show discrepancies between the MDS and conceptual solutions. We opted to map the conceptual solution onto the 11-cluster solution for comparison purposes as it aligned best with the conceptual solution. The statements for each of the constructs listed in Fig. 1 are found in Table 1.

**Table 1:** Conceptual solution of the physical activity parenting practice statements (*n* = 77) sorted by the experts (*n* = 24)

Construct	Number	Abbreviated statement
Neglect/Control		
Permissive	15	Child has a television in bedroom*
	40	Allow child to watch TV or play video/computer games whenever s/he wants to*
	49^a^	Allow child to be less active when on vacation*
	77	Allow child to skip physical activity or sports when s/he wants to
Pressuring	4	Complain to or make child feel bad for not exercising
	9	Tell child that spending time in front of a screen is not good for his/her health/eyesight/weight*
	23	Pressure child to try harder at sports or his/her physical activity
	26^a^	Punish child if s/he is sedentary instead of being active (i.e. no snacks or take away TV/computer privileges)*
	34	Tell my child that s/he needs to exercise so that s/he can lose weight
	36	Show child that you are angry when s/he does not participate in regular physical activity or exercise
	42	Remind/nag child to exercise or be physically active
	43	If child says ‘I don’t feel like walking or bicycling there,’ try to get him/her to do this anyway
	51	Criticize or tell child s/he is not good when doing certain sports
	61^a^	Have a rule ‘If you want a treat, you need to exercise’
	76	Punish child by not allowing him/her to take part in physical activity or sports
Autonomy Support		
Encouragement	2^a^	Tell child that physical activity will make him/her look good
	5	Tell child that physical activity or vigorous exercise is good for his/her health and will make him/her feel good
	7^a^	Show child examples of role models (i.e. people who are active) to encourage him/her to be active
	12	Encourage child to participate in physical activity or play sports (./in his/her free time)
	16	Encourage child to be less sedentary*
	27	Encourage child to be active for at least 60 min per day
	29^a^	Try to encourage child to do physical activities by telling s/he will make new friends
	41^a^	Encourage active video games as a way to be active indoors
	54	Encourage child to walk, bike or use resources (park or community center) in neighborhood to be active
	55	Tell child s/he is doing well in physical activities or sports
	65^a^	Get child to be physically active by telling how much fun the activity is
Guided choice	10	Allow child to choose whether s/he participates in sports or vigorous physical activity in free time
	11	Negotiate with child on how much physical activity/sports s/he does
	28	Negotiate with child on how much TV/video/DVD s/he is allowed to watch*
	45	Provide child with physical activity options from which my child can choose
Involvement	8	Show an interest in child’s sports by talking about his/her activities
	17	Involve child in active chores and yard work around the house
	25	Go to child’s sports or physical activities and watch child participate
	31	Watch sports with child, talk about sports with child, and take child to sports games, to encourage participation in physical activity
	50	Involved in child’s activities (e.g., coaching activities, watching child play)
	52	Spend time teaching child how to play a sport or do certain physical activities
Praises / Rewards	14^a^	Tell child that you like it when s/he is physically active
	20	Reward child for exercising
	75	Praise child for being physically active or for participating in sports
Structure		
Co-participation	6	Practice active habits with child (e.g. parking far from the door, taking the stairs)
	24	Play sports or active games with child
	48	Invite child to join your exercise or do something active with you
	57	Go for walks with child
	71	Use sport/physical activity as a form of family recreation (e.g., going on bike rides together, hiking, skating)
Expectations	35	Make sure child uses active transportation when going places close to home (e.g. walking, biking)
	58	Limit the amount of time child spends [sedentary activity] on weekend/weekday [playing computer games, watching TV, watching videos, electronic games, video games, on the phone]*
	64^a^	Make child responsible for taking the dog for a walk and/or playing with the dog
	68	If the weather is nice, child knows that s/he is expected to play outside
	73	Make sure child is physically active at least 60 min per day
	74	Have a rule that child must participate in active sports or physical activities
Facilitation	18	Buy/provide physical activity or sports equipment for child
	22	Take child to the park, playground, or places that s/he can be physically active
	39	Help child find ways to reduce his/her sedentary habits
	47	Store child’s active toys/sports equipment in a place that is easily accessible
	63	Enroll child in sports and physical activity programs
	67	Try to make physical activity into a fun game to get child more active
	69	Arrange for child to be with friends in order to be active with them
	70^a^	Encourage competition or set challenges (e.g., walking a certain distance) during activities to get child more active
Modeling	46	Child sees you being sedentary or is sedentary with you*
	60	If you would like to watch TV/video/DVD, you restrain yourself because of the presence of child*
	72	Use own active behavior to encourage child to be physically active
Monitoring	44	Keep track of the amount of physical activity or exercise child gets
	53	Keep track of the amount of time child spends in front of screens (e.g television, computer)*
Restriction for safety/academic concerns	1	Restrict child’s outdoor activities because neighborhood is not safe
	13	When child plays outside, s/he must be supervised
	21	Don’t allow child to play on community or sports teams (./so s/he can concentrate on schoolwork)
	30	Have rules that child is not allowed to walk to the neighborhood park alone
	37^a^	Have a rule that child must do homework before s/he is able to exercise or be physically active
	38	Don’t allow child to play outside in the street after dark or after a certain time
	56^a^	Prohibit child from playing certain sports
	59	Restrict some physical activities because afraid child will be hurt
	62	Restrict the amount of time child spends playing outside
	66	Restrict [activity type] inside the house [active play, ball games, running, riding tricycle/scooter]
Drop	32	Reward child for good behavior with TV, DVD, or computer time – Drop because the behavior is unspecified and need practices that are more specific)*
	19	Do not enroll child in physical activities that are too expensive – Drop because (Capture socio-economic issues which does not fit with other items dropped from clustering but can be included as a single item)
	3	Enroll child in too many activities leaving no time for free play (not specific to physical activity)
	33	Try to get child to be active (e.g. playing tag, biking, dancing) instead of watching TV or playing video games (Drop because how parent achieve this is unspecified)

*Practices related to sedentary behaviors were omitted from the operational definition as the focus was on physical activity

^a^Key practices that were identified from parent responses [22].

In comparison to the MDS solution, the conceptual solution produced by the experts modified the boundaries of some clusters as the content of some statements seemed more conceptually related to nearby constructs. Such examples can be found in constructs E (co-participation), F (involvement), G (facilitation), H (expectations), J (pressuring), and K (praises/rewards). While it is important to consider the proximity of the statements, reshaping of the statistical clusters occurred when it made theoretical sense to do so. Reshaping predominantly occurred when a parenting practice statement was thought to better fit conceptually with the operational definition of a neighboring cluster and both the larger team of investigators and experts agreed with this decision. For example, the parenting practice statement number 75 “Praise child for being physically active or for participating in sports” was merged with the praises/rewards construct as the construct already included a practice statement related to praise, namely number 14 “Tell child you like it when s/he is physically active”.

In the MDS statistical solution, the monitoring and permissive constructs were combined (see clusters A and B, respectively); however, after examining the content of this combined cluster it was determined these two concepts should not be combined. Although merging of clusters A (monitoring) and B (permissive) appeared early in the clustering process (i.e., at the 21st cluster solution), it did not make conceptual sense to regroup them. As a result the cluster was separated.

Construct G, labelled facilitation for physical activity emphasizes the ways in which parents support participation in physical activity including financial assistance, provision of material goods, and planning and facilitating physical activity. However, at the bottom of cluster G there are three practices which were not added to this cluster as they fit better with nearby clusters (8 and 25 fit better with involvement and 7 with encouragement). As they fall in between these two clusters, it suggests less agreement as to what fits with the parental involvement and encouragement clusters.

The MDS12-cluster solution shows four parenting practice statements grouped as a thin and long cluster (statements 9, 14, 20, and 34 located on the right side of the map). Grouping of these four statements occurred at the 13-cluster solution and in prior solutions, statements 14 and 20 were grouped together and statements 9 and 34 were grouped together. Evaluation of the map revealed that statements 14 “Tell child that you like it when s/he is physically active”, 20 “Reward my child for exercising”, and 75 “Praise child for being physically active or for participating in sports” refer to praises/rewards and were regrouped under construct K. In contrast, statements 9 “Tell child that spending time in front of a screen is not good for his or her health/eyesight/weight” and 34 “Tell child that s/he needs to exercise so that s/he can lose weight” relate to pressuring the child to be active for health concerns and were grouped with construct J (pressuring).

Although some practices regrouped into some clusters, we opted to delete some of them because they were either too vague or they measured irrelevant concepts (statements 3, 19, and 32 were dropped). Statement 3 “enroll my child in too many activities leaving no time for free play” was deleted from construct H (expectations) as this concept can have both positive and negative implications for a child’s sustained participation in physical activity (increase their physical activity but decrease their sense of autonomy). Statement 19 “Do not enroll child in physical activities that are too expensive” was deleted from construct I (restriction for safety or academic concerns) because it captures socio-economic issues. While cost is a restriction to enrollment and participation in physical activity, it is preferable to measure this aspect separately as it affects only certain families. Finally, statement 32 “reward my child for good behavior with TV, DVD, or computer time” was deleted from construct H (expectations) as the “good behavior” was not specified and items that capture this concept should be regrouped under construct K (praises/rewards).

Finally, while most experts endorsed the conceptual solution which includes 12 constructs, some identified potential overlaps with some constructs – including co-participation with involvement and encouragement with praises/rewards. We opted to keep these constructs separate at this stage, while acknowledging that further psychometric work will enable us to shed light on whether such an operationalization is supported, or whether further refinement is needed. In addition, many experts indicated that the statements related to sedentary behaviors should be excluded from the physical activity parenting practice item bank. These statements are highlighted in Table 1 and our construct definitions have eliminated these statements from the operational definitions.

### Categorizing the physical activity parenting practices into a recent parenting taxonomy

**Fig. 2:**
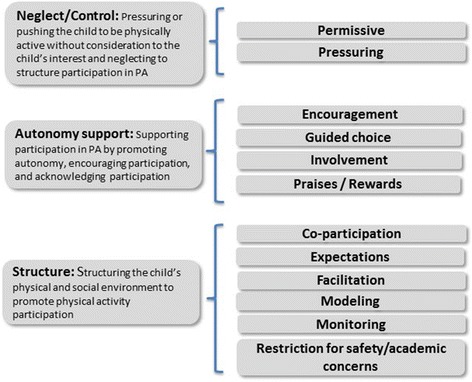
Categorizing the physical activity (PA) parenting practices into current parenting taxonomy

Figure 2 shows how each of the physical activity parenting practice constructs can be grouped into higher-order domains of parenting: neglect/control, autonomy support, and structure. Davison et al.’s [14] and Vaughn et al.’s [30] papers informed this categorization. Definitions for these domains are shown in Fig. 2 with Table 2 providing the operational definitions for each construct.

**Table 2:** Definition of physical activity parenting practice constructs

Domain/Construct	Definition
Neglect/Control	
• Permissive	Parent does not guide their child’s behaviors and allows them to decide whether they engage in physical activity
• Pressuring	Parent criticizes, nags, forces, pressures, punishes, or uses threats to get their children to be physically active
Autonomy Support	
• Encouragement	Parent suggests or encourages child to be physically active by explaining the reasons for being active, highlighting role models or provides positive verbal reinforcement for doing so
• Guided Choice	Parent promotes independence in decisions related to physical activity by providing child with options or by negotiating with the child
• Involvement	Parent demonstrates an interest in the child’s participation in physical activity or sports by watching child participate in his/her physical activity or sports, talking about his/her physical activities, teaching child new skills, and volunteering/coaching in child physical activity or sports
• Praises/Rewards	Parent positively reinforces participation in PA by verbally praising their child or acknowledging their participation without coercing their participation
Structure	
• Co-participation	Parent engages in physical activity with their child
• Expectations	Parent sets clear expectations about physical activity as to when and how much physical activity the child should do
• Facilitation	Parent positively supports child physical activity by getting them involved in activities through enrollment or taking them to places to be active, and by supporting their physical activity (financial assistance, provision of equipment, services such as transportation and planning physical activities)
• Modeling	Parent models an active lifestyle
• Monitoring	Parent tracks child involvement in physical activity
• Restriction for safety/academic concerns	Parental concerns about safety and academic performance results in limiting child involvement in physical activity

## Discussion

To minimize inconsistencies in measures of physical activity parenting practices, [11, 14, 16, 18] this study utilized concept mapping methods to aggregate input from experts to identify constructs of parenting practices. Expert sorting of 77 parenting practice statements identified from a review of the literature and from interviews with parents, resulted in identifying 12 constructs which are presented using the three main domains of general parenting practices, namely neglect/control, autonomy support, and structure. The neglect/control domain includes two constructs: permissive and pressuring parenting practices. The autonomy support domain includes four constructs: encouragement, guided choice, involvement in child physical activities, and praises/rewards to encourage their children’s physically activity. Finally, the structure domain includes six constructs: co-participation, expectations, facilitation, modeling, monitoring, and restricting physical activity for safety or academic concerns.

The constructs were categorized under the three main domains of parenting which have recently been used to classify food and physical activity parenting practices [14, 30] and integrate terms from developmental psychology to characterize parenting more broadly [9, 28, 29]. The neglect/control domain partially aligns with Baumrind’s definition of control which reflects the “claims that parents make on children to become integrated into society through behavior regulation, direct confrontation, and maturity demands (behavioral control), and supervision of children’s activities” [28]. In our framework, the neglect/control domain includes the coercive components of Baumrind’s definition with some aspects of control classified under structure to highlight that some level of control is necessary to set the proper environment for children to be physically active. As a result, both expectations and monitoring were classified under structure as they provide necessary structure and boundaries to the child. This aligns with Grolnick and Pomerantz’s [31] conceptualization of control which suggests regrouping the dominating and pressuring parenting practices under control; whereas, control practices that offer guidance to the child should be regrouped under structure.

Darling and Steinberg’s operationalized control (demandingness) in terms of “the parent’s willingness to act as a socializing agent” [9]. This prompted us to classify being permissive with control to capture *a lack* of “willingness to act as a socializing agent” as measuring the opposite end of this continuum (neglect/control). Both constructs are independent of each other but are regrouped together as they capture less desirable parenting practices.

The autonomy support domain aligns with Baumrind’s definition of responsiveness which includes “the extent to which parents foster individuality and self-assertion by being attuned, supportive, and acquiescent to children’s requests: it includes warmth, autonomy support, and reasoned communication” [9, 28]. Finally, the structure domain aligns with current definitions that focus on structuring the child’s environment [29] to achieve specific childrearing outcomes.

Our classification differs slightly from Davison’s [14] physical activity and Vaughn’s [30] food parenting practice classifications as: 1) we utilized different terminology to refer to one of the domains of parenting, where our classification refers to what others have termed demandingness or control as neglect/control; and 2) we classified expectations under structure which follows Vaughn’s classification and Grolnick and Pomerantz conceptualization of control [30, 31]. Importantly regrouping the constructs into the three broad domains of parenting shown in Fig. 2 does not imply that the constructs measure a higher order factor. For example, the extent to which the constructs within the autonomy support domain should be examined together or separately will depend on the psychometric properties of this domain – whether the four constructs measure a higher order factor or not. Based on our concept mapping results, the encourage and praises/rewards constructs may turn out to be highly correlated as they are proximally located on the point map (see Fig. 1). However, the remaining constructs, namely involvement and guided choices, will likely measure independent dimensions. Instead, the classification is useful as it highlights the need to examine overall profile of parenting and that this complexity needs to be accounted when one examine their impact on children health behaviors.

The exploratory nature of this analysis means that it is possible for some statements to be misclassified. However, any misclassified statements did not result in identifying new constructs which provided some validity for the concept mapping results. Importantly, the analytical process used in this paper identified the main constructs and provided some examples of parenting practices that fit under these constructs. In the creation of the item bank, it will be important to maintain items that match these operational definitions, but to consider how theories and models of health behavior can inform the operationalization of these constructs (e.g., social support models, [32] self-determination theory, [33] social cognitive theories, [34] socio-ecological models, [35] among others). Importantly, once we collect data among parents, we will be able to refine measures of these constructs and analyze whether all the constructs are independent or whether there is some overlap among them as suggested by some of the experts.

Enabling comparisons across studies is an essential step to elucidate the mechanisms through which parents can influence children’s physical activity. This study will provide the foundation for operationalizing measures of physical activity parenting practices which can be used in observational and/or intervention studies. The constructs identified from the concept mapping analysis will provide the foundation for developing an item bank calibrated with Item Response Modeling [36] supported with computerized adaptive testing which will standardize the measurement of parenting practices while allowing researchers some flexibility in selecting items of interest [15]. Specifically, utilizing the item bank with computerized adaptive testing will allow physically activity researchers to tailor the measurement of parenting practices and reduce the burden of completing lengthy questionnaires. This process works by first having participants answer select items for a specific physical activity parenting construct, with their responses determining which items they receive next. The computer stops administering items for a specific physical activity parenting construct when the parental score on a given construct can be estimated with enough precision. Within intervention studies, baseline assessments of certain constructs which are not often used by parents, such as guided choices, [22] could be measured with fewer items at baseline and presumably with more items in the follow-up if the physical activity intervention focused on this aspect of parenting. Interestingly, the procedure allows some flexibility of adding new items for a given physical activity parenting construct, and as long as the researcher utilizes some of the items that have been pre-calibrated in the item bank, they will be able to compute a score for a given construct that can be compared across studies. While the field of physical activity has not yet taken advantage of these advanced psychometric methods, there are successful examples in the fields of outcomes research (e.g., to measure quality of life) which can serve as models for the field of physical activity (see www.nihpromis.org/ NIH PROMIS initiative) [36].

The concept mapping analysis identified a number of central constructs that ought to be included in measures of physical activity parenting practices. It is possible that constraining the number of statements provided to the experts as well as the selection of specific statements could have biased the types and numbers of constructs identified. Furthermore, the statements provided to the experts were in some instances more generic than the original items or parent responses. As a result, these small nuances were not captured and likely yielded broader constructs or can explain why some statements were not clearly located in the clusters to which they conceptually belong. While it is likely that other relevant constructs have not been captured through our concept mapping analysis, the ones identified likely need to be incorporated in future research and provide a basis for measuring physical activity parenting practices.

## Conclusions

The concept mapping analysis engaged experts in re-conceptualizing measures of physical activity parenting which provided an initial roadmap for developing an item bank that captured 12 key physical activity parenting constructs.

## Abbreviations

AWT: Andrew W. Tu
DVD: Digital Video Disc
IRM: Item response modeling
ISBNPA: International Society of Behavioral Nutrition and Physical Activity
LCM: Louise C. Mâsse
MDS: Multidimensional Scaling
MRB: Mark R. Beauchamp
PA: Physical Activity
PROMIS: Patient Reported Outcomes Measurement Information System
SOH: Sheryl O. Hughes
TB: Tom Baranowski
TMO: Teresia M. O’Connor
TV: Television
US: United States
